# Stoichiometry of carbon, nitrogen, and phosphorus released from the leaf litter of various temperate tree species

**DOI:** 10.1002/ece3.10372

**Published:** 2023-07-25

**Authors:** Pei‐Chi Ho, Suzuna Nakajima, Jotaro Urabe

**Affiliations:** ^1^ Graduate School of Life Sciences Tohoku University Sendai Japan

**Keywords:** aquatic food web, ecological stoichiometry, leaf litter, terrestrial subsidy

## Abstract

Dissolved organic matter and inorganic nutrients released from forest leaf litter through leaching are the important energy and nutrient sources that support the production of aquatic food webs. Leaf litter‐derived dissolved organic carbon (DOC) is a critical energy source for aquatic heterotrophic microbes, and inorganic nitrogen and phosphorus can enhance primary production. In this study, we experimentally measured the release efficiencies and amounts of DOC, total dissolved nitrogen (TDN), and total dissolved phosphorus (TDP) of the leaf litter from 11 temperate tree species by soaking the leaf litter in water for 28 days. We found that the maximal release efficiency (% of element released per estimated mass of the element) was the highest for P and lowest for N. These efficiencies were species‐specific. Additionally, the DOC:TDP, DOC:TDN, and TDN:TDP ratios varied among the leachate of different leaf litter species. DOC:TDP increased with the C:P ratio in leaf litter biomass but is considerably lower; TDN:TDP was lower than the N:P ratio in leaf litter biomass as well; DOC:TDN ratio was higher than the C:N ratio in leaf litter biomass. These results suggest that the ratios of DOC to dissolved N and P nutrients released into water are related to, but not the same as, the stoichiometry of leaf litter biomass. Based on these findings, we concluded that changes in the vegetations with different leaf litter stoichiometry can alter the relative importance of detrital and grazing food chains in aquatic ecosystems.

## Graphical abstract

Dissolved C, N, and P from forest leaf litter are important energy and nutrient resources to freshwater food webs. We investigated the release efficiency of dissolved C, N, and P of 12 of leaf litter species and the stoichiometric traits of leaf litter that affect release efficiency. Based on the investigation, we concluded that changes in the vegetations with different leaf litter stoichiometry can alter the relative importance of detrital and grazing food chains in aquatic ecosystems.

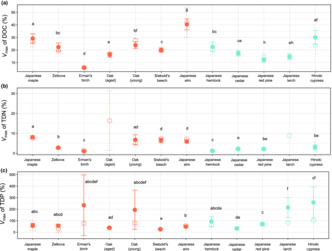

## 1 INTRODUCTION

As in other ecosystems, the aquatic food web comprises detrital and grazing food chains (Hairston & Hairston, 1993; Lindeman, 1942). Terrigenous organic matters, such as leaf litter, are considered important energy and nutrient sources that sustain the food webs in ambient freshwater ecosystems. Organic carbon in the leaf litter can serve as a substrate for heterotrophic microbes in lakes and rivers (Carpenter et al., 2005; Cole et al., 2011; Hiltunen et al., 2017), thereby supporting the detrital food chains (Hirama et al., 2022; Tanentzap et al., 2014, 2017). When dissolved nitrogen and phosphorus are released from leaf litter and enter into aquatic ecosystems, they can be used for primary production by phytoplankton, which are the base of grazing food chains (Hirama et al., 2022; Kissman et al., 2017). Consequently, leaf litter can stimulate both the detrital and grazing food chains in aquatic ecosystems. However, the relative importance of the leaf litter in mass flow along the detrital and grazing chains may vary among the leaf litter species if the release efficiencies and rates of dissolved organic carbon (DOC), total dissolved nitrogen (TDN), and total dissolved phosphorus (TDP) differ among leaf litter species (Hirama et al., 2022).

The C, N, and P contents of leaf litter differ considerably among tree species (Schreeg et al., 2013; Yan et al., 2022). Furthermore, the release of nutrients from the leaf litter of coniferous species is lower and slower than from that of broadleaf species on soil (Usman, 2013). Leaf litter from tree species with high lignin contents or high C to nutrient ratios generally decomposes more slowly and releases lower amounts of dissolved nutrients (Berg & McClaugherty, 1989; Osono & Takeda, 2004). Indeed, many studies have shown that the decomposition rate of the leaf litter differs among tree species, suggesting that the release efficiencies of elements, that is, fractions of DOC, TDN, and TDP in the C, N, and P contents of the leaf litter, respectively, differ among tree species. In addition, the senescence of leaf litter may change the elemental composition and release of elements. Fellman et al. (2013) suggest that as leaf litter ages for more than 2 months, the DOC and N released from leaf litter gradually decreased.

P in leaf litter mainly exists as water‐soluble orthophosphate and inositol phosphate (Chapin et al., 1990; Yang et al., 2017). Therefore, the release efficiency of P is high, and P is released rapidly during the early stages of litter leaching and decomposition (McComb et al., 2007; Pourhassan et al., 2016; Schreeg et al., 2013). In contrast, C and N in leaves exist mainly in structural components (Chapin et al., 1990), such as lignin and cellulose, which are insoluble in water and difficult for most microbes to decompose (Johansson, 1995; Kögel‐Knabner, 2002). Additionally, most of water‐soluble N in leaves are reabsorbed back to the trunk before defoliation in many species (Pate, 1980). The fact suggests that the release efficiency of TDP from leaf litter is higher than those of DOC and TDN. However, few studies have examined the release efficiencies of these elements from the leaf litter soaked in water, which simulates the leaching and decomposition of leaf litter in freshwater ecosystems. Consequently, information regarding the tree species–specific release rates of DOC, TDN, and TDP of leaf litter in water is limited. If the release rates of these elements differ among the leaf litter of different tree species, it is likely that changes in the forest vegetation of a watershed can alter the relative importance of detrital and grazing food chains in aquatic ecosystems.

Therefore, in the present study, we investigated the C:N:P stoichiometry and the DOC, TDN, and TDP release efficiency of leaf litter of various tree species at the early stage of decomposition in a freshwater environment to examine whether changes in the watershed vegetation affect relative contributions of detrital and grazing food chains sustaining aquatic food webs. To this end, we soaked the leaf litter from 11 temperate tree species in aerated water and quantified the DOC, TDN, and TDP released over 28 days. To investigate the effect of leaf litter aging on the release of DOC, TDN, and TDP, we selected oak (*Quercus serrata*), which is one of the most common and important broadleaf *Quercus* species in temperate forests (Bölöni et al., 2021), and collected greenish (presumably young) and brownish (presumably aged) leaf litter for the experiment. Subsequently, we examined the following four uncertainties: does the release efficiency of leaf litter differ among DOC, TDN and TDP; are these release efficiencies species‐specific; what factors or elemental components in the leaf litter determine these release efficiencies; does leaf litter senescence affect DOC, TDN, and TDP release?

## 2 MATERIALS AND METHODS

### 2.1 Collection and elemental content measurement of leaf litter

**FIGURE 1:**
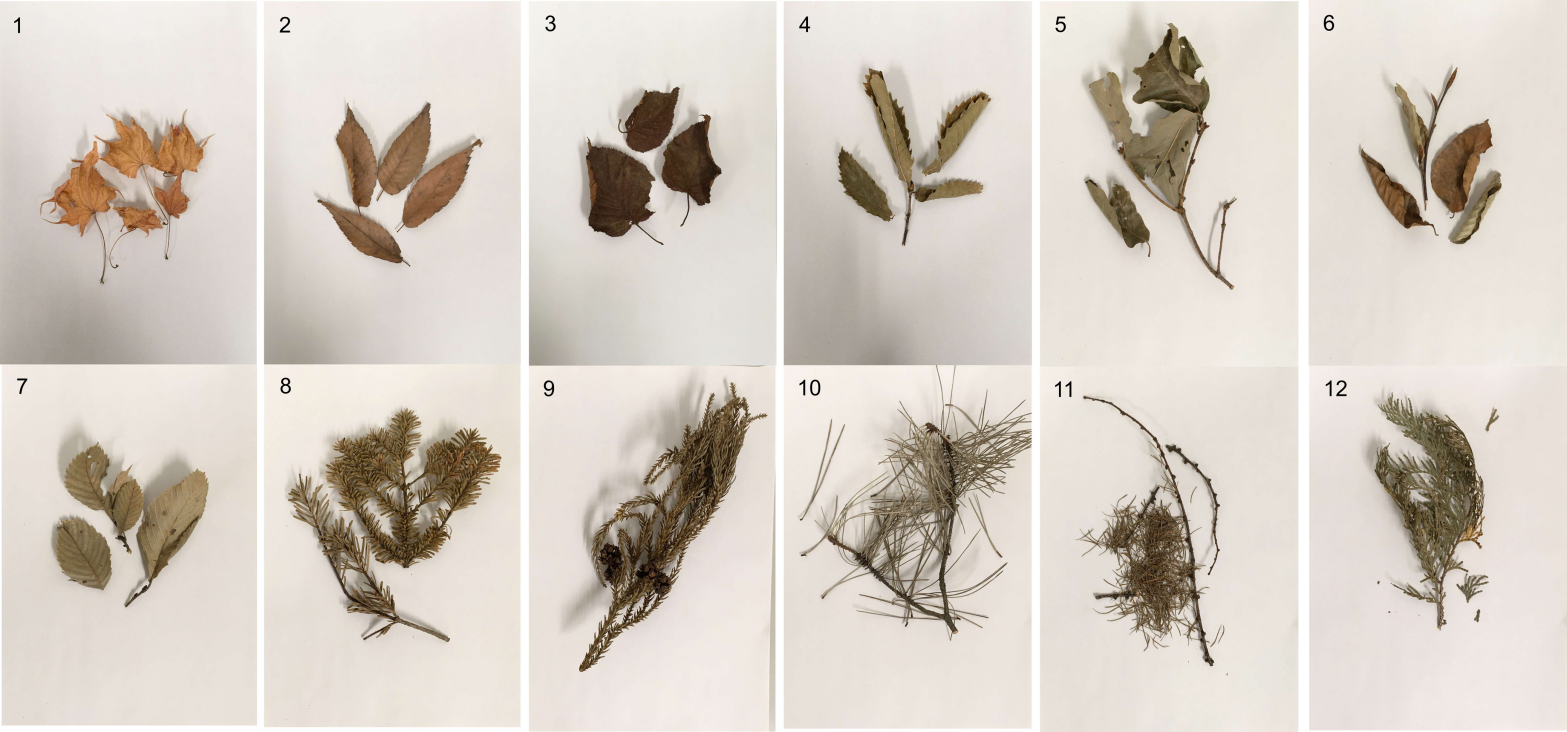
Images of 12 types of leaf litter collected. 1. Japanese maple (*Acer amoenum* var.amoenum), 2. Zelkova (*Zelkova serrata*), 3. Erman's birch (*Betula ermanii*), 4. Oak (aged) (*Quercus serrata*), 5. Oak (young) (*Quercus serrata*), 6. Siebold's beech (*Fagus crenata*), 7. Japanese elm (*Ulmus davidiana* var. japonica), 8. Japanese hemlock (*Tsuga diversifolia*), 9. Japanese cedar (*Cryptomeria japonica*), 10. Japanese red pine (*Pinus densiflora*), 11. Japanese larch (*Larix leptolepis*), and 12. Hinoki cypress (*Chamaecyparis obtusa*).

We examined 11 tree species commonly found in the mountainous areas of northeast Japan. These shed leaves were collected at Zao (N38°7'19.1994", E140°27'3.5994") on October 3, 2020, at Kawatabi (N38°44'41.9994", E140°45'25.2") on October 15, 2020, and at Aobayama (N38°15'34.4", E140°50'13.1994") on November 19, 2020 in Miyagi Prefecture, Japan (Figure 1; Table 1). All leaves were collected on top of other leaf litter on the ground, and therefore did not directly come into direct contact with the soil. In oak, we collected greenish and aged leaf litter. The greenish leaf litter appeared to be freshly shed and was therefore treated as young leaf litter, whereas the brownish leaf litter appeared to be withered and was therefore treated as aged leaf litter (Figure 1, panels 4 and 5). Thus, we examined a total of 12 types of leaf litter.

**TABLE 1:** Stoichiometry of leaf litter collected on October 3, 2020 in Zao, on October 15, 2020 in Kawatabi, and on November 19, 2020 in Aobayama in Miyagi Prefecture, Japan, showing the mean and standard deviation (±values) of the elemental contents with the number of measurements and the C/N, C/P, and N/P molar ratios calculated from the mean of the C, N, and P contents.

No.	Tree species				Sampling site			Litter stoichiometry					
	Common name	Species name	Tree type	Leaf type	Area	Latitude	Longitude	C (μg/mg), Mean ± SD (*n*)	N (μg/mg), Mean ± SD (*n*)	P (μg/mg), Mean ± SD (*n*)	Molar C/N	Molar C/P	Molar N/P
1.	Japanese maple	*Acer amoenum* var.amoenum	Broadleaf	Deciduous	Aobayama	N38°15'32.4"	E140°50'13.1994"	403.40 ± 8.07 (4)	9.56 ± 1.10 (4)	0.29 ± 0.046 (5)	49.70	3629.67	73.71
2.	Zelkova	*Zelkova serrata*	Broadleaf	Deciduous	Aobayama	N38°15'32.4"	E140°50'13.1994"	389.16 ± 3.82 (4)	13.18 ± 2.07 (4)	0.44 ± 0.029 (5)	35.00	2290.50	66.51
3.	Erman's birch	*Betula ermanii*	Broadleaf	Deciduous	Zao	N38°7'44.3994"	E140°27'46.7994"	489.34 ± 3.79 (4)	29.55 ± 1.48 (4)	0.73 ± 0.026 (5)	19.36	1724.26	89.24
4.	Oak (old)	*Quercus serrata*	Broadleaf	Deciduous	Zao	N38°7'19.1994"	E140°27'3.5994"	458.88 ± 8.63 (4)	19.10 ± 1.59 (4)	1.11 ± 0.19 (5)	28.21	1066.16	38.03
5.	Oak (young)	*Quercus serrata*	Broadleaf	Deciduous	Kawatabi	N38°44'41.9994"	E140°45'25.2"	460.78 ± 3.10 (4)	28.18 ± 0.86 (4)	3.20 ± 0.61 (3)	19.09	371.21	19.46
6.	Siebold's beech	*Fagus crenata*	Broadleaf	Deciduous	Kawatabi	N38°44'41.9994"	E140°45'25.2"	538.27 (1)	16.27 (1)	6.02 ± 0.60 (3)	38.59	230.81	5.98
7.	Japanese elm	*Ulmus davidiana* var. japonica	Broadleaf	Deciduous	Kawatabi	N38°44'41.9994"	E140°45'25.2"	401.98 ± 5.08 (4)	20.38 ± 0.71 (4)	9.79 ± 2.96 (3)	23.03	105.98	4.60
8.	Japanese hemlock	*Tsuga diversifolia*	Coniferous	Evergreen	Zao	N38°7'19.1994"	E140°27'3.5994"	500.32 ± 3.58 (4)	16.02 ± 1.36 (4)	0.78 ± 0.13 (5)	36.62	1651.98	45.34
9.	Japanese cedar	*Cryptomeria japonica*	Coniferous	Evergreen	Kawatabi	N38°44'41.9994"	E140°45'25.2"	489.19 ± 7.47 (4)	13.67 ± 0.52 (4)	1.12 ± 0.42 (5)	41.80	1131.20	27.09
10.	Japanese red pine	*Pinus densiflora*	Coniferous	Evergreen	Kawatabi	N38°44'41.9994"	E140°45'25.2"	498.30 ± 6.56 (4)	19.69 ± 3.09 (4)	1.26 ± 0.09 (3)	30.06	1016.87	34.44
11.	Japanese larch	*Larix leptolepis*	Coniferous	Deciduous	Kawatabi	N38°44'41.9994"	E140°45'25.2"	453.12 ± 7.34 (4)	23.50 ± 1.87 (4)	1.52 ± 0.29 (3)	22.58	768.75	34.17
12.	Hinoki cypress	*Chamaecyparis obtusa*	Coniferous	Evergreen	Kawatabi	N38°44'41.9994"	E140°45'25.2"	488.57 ± 2.12 (4)	18.68 ± 1.65 (4)	1.90 ± 0.31 (3)	30.69	662.66	21.72

The leaf litter was dried at room temperature (approximately 20°C) in paper bags until it was used for measuring its C, N, and P contents. Prior to the experiment, the C and N contents in the leaf litter were measured using an elemental analyzer (2400 Series II CHNS/O Analyzer; Perkin Elmer). For estimating the P content in the leaf litter, the weighed amount of dry leaf litter was first combusted (420°C) for 2 h to turn the leaf litter into ash, which was suspended in 10 mL of distilled water. The mixture of ashes and distilled water was autoclaved with potassium persulfate for 30 min to oxidize organic phosphorus compounds and the total orthophosphate concentration was measured by ascorbate‐reduced molybdenum blue method (Menzel & Corwin, 1965; Murphy & Riley, 1962).

### 2.2 Experiment

Prior to the leaching incubation experiment, 1 L experimental bottles and ventilation equipment were washed with phosphorus‐free detergent, rinsed with 0.1 M HCl solution, and then rinsed with distilled water. We cut the dry leaf litter using scissors to mimic the initial decomposition process of detritivores and immersed the cut dried leaf litter into 1 L experimental bottles containing 800 mL distilled water. Two replicates of the leaching bottles were set up for each leaf litter species. These bottles were aerated and placed in dark incubators at 20°C for 28 days. The dry weight of leaf litter used for leaching incubation was 7–385 mg, depending on the tree species: for tree species with low leaf litter P, a larger amount of leaf litter was added into experimental bottles to ensure that the P in leachate would be of the same concentration level, thus minimizing and equalizing measurement errors.

During the experiment, we shook the bottles manually to mix the leachate once a day. On Days 1, 3, 7, 14, 21 and 28, we sampled 50 mL of the supernatant of the leachate after all leaf litter had settled down to the bottom of the bottle and did not refill the bottle with new distilled water. These samples were used to measure the DOC, TDN, and TDP in the leachate. DOC and TDN in the litter leachate were measured using a TOC/TNb analyzer (multi N/C 3100; Analytik Jena GmbH). For TDP measurements, 10 mL of leaf litter leachate was autoclaved with potassium persulfate and measured using the ascorbate‐reduced molybdenum blue method.

### 2.3 Estimation of release efficiencies of leaf litter C, N, and P

We calculated the percentage of release relative to the total content of an element in the leaf litter as the release efficiency (%):

(1) $$V_{E,t}=\frac{D_{E,t}}{E}\times 100\%,$$

where *D*_*E*,*t*_ is the concentration of dissolved element *E* (DOC, TDN, or TDP) at day *t* and *E* is the elemental concentration (C, N, or P, respectively) of the leaf litter added into the bottle. *E* was calculated as follows:

(2) $$E=\frac{\text{Litter weight}\;\left(\mathrm{mg}\right)\times \text{Litter}\;E\;\text{mass}\;\left(\mathrm{\mu g}/\mathrm{mg}\right)}{0.8\;\left(\text{liter}\right)}.$$

We determined the maximal release efficiency (*V*_max_) and half saturation time (*k*) of an element *E* by fitting the release efficiency *V*_*E,t*_ and sampling day *t* of pooled data from two replicates of each type of leaf litter to the Michaelis–Menten equation as follows:

(3) $$V_{E,t}=V_{\max E}\frac{t}{k+t}.$$

The fitting was performed by nonlinear least‐squares regression using the *nls* function in the statistical package of R ver. 4.2.2 (R Core Team, 2022). The coefficient of determination (*r* ^2^) was estimated through correlation coefficient analysis between the observed *V*_*E*,*t*_ and estimated *V*_*E*,*t*_ from Equation 3 (Figures S1–S3).

We further calculated the maximal total release amount (*TRA*) of dissolved element *E* for the 28‐day experiment using *V*_max*E*_ and *E* mass in leaf litter:

(4) $${\mathrm{TRA}}_{E}=V_{\max E}\times \text{Litter}\;E\;\text{mass}\;\left(\mathrm{\mu g}/\mathrm{mg}\right).$$

If *V*_max*E*_ from the Michaelis–Menten fitting was higher than 100%, we used the mean maximal release efficiency of element *E* actually observed in the two replicates during the experiment (Max‐*V*_*E*_) instead for further analyses. To compare the molar C:P, C:N, and N:P ratios of leaf litter and leachate, we also estimated the DOC:TDP, DOC:TDN, and TDN:TDP ratios of *TRA*, that is, *TRA*_C:P_, *TRA*_C:N,_ and *TRA*_N:P_. respectively.

### 2.4 Statistical analysis

We examined the differences in *V*_max_ between the leaf litter of tree species using pairwise Welch's *t*‐test (Welch, 1947) using the variance of these coefficients estimated with the *nls* function. In this test, alpha level for significance (*p* < .05) was adjusted using the Bonferroni correction. We examined the effects of leaf litter elemental contents and ratios on *V*_max_ (or Max‐*V*) and *TRA* using least‐squared linear regression analyses.

## 3 RESULTS

### 3.1 Elemental content of the leaf litter

The C, N, P content and molar C:N, C:P, and N:P ratios varied considerably among the 12 types of leaf litter (Table 1). The C, N, and P mass ranged from 389.16 to 538.27 μgC/mg dry litter mass, 9.56 to 29.56 μgN/mg dry litter mass, and 0.29 to 9.79 μgP/mg dry litter mass, respectively. The molar C:N ratio showed a 2.6‐fold variation while the C:P and N:P ratios showed a 34‐ and 20‐fold variations among the leaf litter, respectively.

### 3.2 Release efficiency of DOC, TDN, and TDP

**FIGURE 2:**
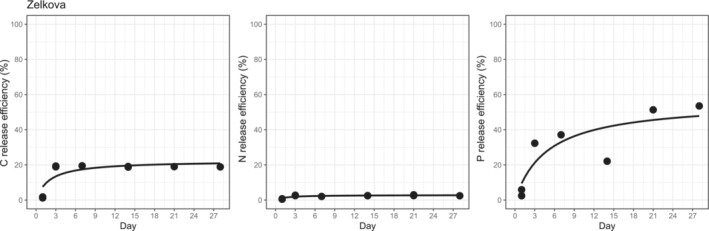
Examples of the Michaelis–Menten equation fit to the release efficiencies of C, N, and P (*V*_*E*_) during the 28‐day incubation experiment using zelkova leaf litter.

**FIGURE 3:**
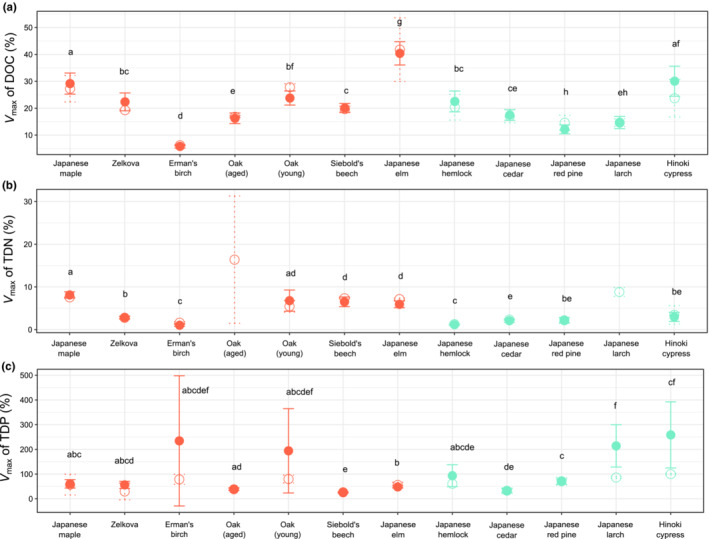
Maximal release efficiency (*V*_max_) and its standard deviation for dissolved organic carbon (DOC) (a), total dissolved nitrogen (TDN) (b), and total dissolved phosphorus (TDP) (c) estimated from fitting the Michaelis–Menten equation (solid circles and lines) and from the observed maximal release (Max‐*V*_*E*_) during the 28‐day leaching experiment (hollow circles and dotted lines). The standard deviation of *V*_max_ of the Michaelis–Menten function fitting was estimated based on the regression coefficient, and the standard deviation of Max‐*V* was calculated from the observed maximal *V*_*E*_ values of the two replicates during the leaching experiment. Red circles denote broadleaf and aqua blue circles denote coniferous leaf litter. Significant differences of *V*_max_ estimated from the Michaelis–Menten equation fitting are labeled with different letters.

The release efficiency (*V*_*E*_) against time was significantly fitted to the Michaelis–Menten equation (*p* < .05) for most of the leaf litter, as shown in the examples of zelkova leaf litter in Figure 2, except for TDN of aged oak (*Quercus serrata*) and Japanese larch (*Larix leptolepis*) leaf litter (Figures S1–S3). The *V*_max_ estimated by fitting to the Michaelis–Menten equation exceeded 100% for TDP in leaf litter of Erman's birch (*Betula ermanii*), young oak (*Quercus serrata*), Japanese larch, and hinoki cypress (*Chamaecyparis obtusa*). The *V*_max_ of TDP was higher than those of DOC and TDN in all the leaf litter examined. However, the half saturation time of release efficiency (*k*) was the shortest for DOC (2.41 days; Table S1), followed by TDN release (3.04 days; Table S1), and was the longest for TDP (16.99 days; Table S1). The *V*_max_ for DOC, TDN, and TDP differed between the leaf litter types (Figure 3). Between broadleaf and coniferous leaf litter, the mean *V*_max_ of DOC (mean *V*_max_ = 22.54% and 19.37%, respectively) and TDN (mean *V*_max_ = 5.20% and 2.10%, respectively) of broadleaf species was higher (*t* = 2.38, *p* = .01 and *t* = 7.44, *p* < .001, respectively). However, no significant difference was detected in *V*_max_ of TDP between the broadleaf and coniferous species. We also examined the maximal release efficiency that was actually observed during the 28‐day experiment (Max‐*V*), which was, on average, 59.74% for TDP across all species, and was also the highest among the elements, followed by that for DOC (20.76%), and the lowest for TDN (5.53%; Table S1).

### 3.3 Leaf litter stoichiometry versus release stoichiometry

**FIGURE 4:**
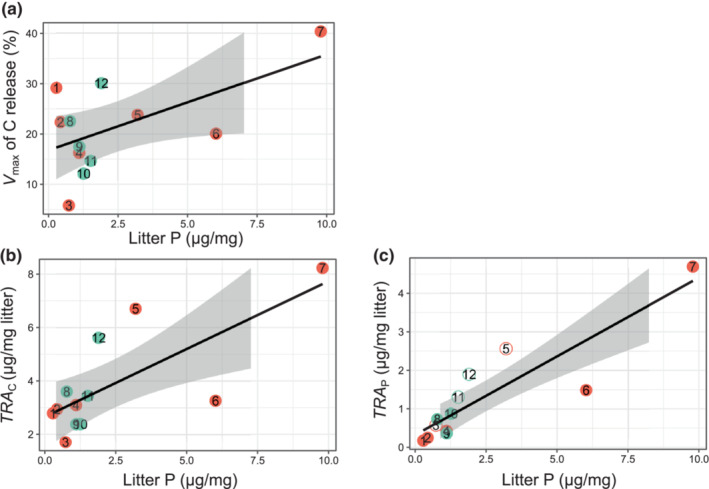
Linear regression between (a) maximal release efficiency (*V*_max_) of dissolved organic carbon (DOC), (b) maximal total release amounts of DOC (*TRA*_C_) and (c) total dissolved phosphorus (TDP) per mg leaf litter dry mass (*TRA*_P_) against P contents in leaf litter. Open circles in subplots denote observed maximal release efficiency (Max‐*V*_*E*_) from the incubation experiments that are estimated for tree species with *V*_max_ > 100% (P release from Erman's birch, oak [young], Japanese larch and hinoki cypress). Numbers signify tree species in the order outlined in Table 1 and Figure 1. Significant linear regression and the estimated 95% confidence interval of the linear fitting are presented as solid lines and shaded areas.

No significant effects of the leaf litter content on *V*_max_ were detected for any of the elements (Figure S4a,e,i; Table S2). However, we found that *V*_max_ of DOC was positively associated with P content in the leaf litter (Figure 4a; Table S2).

We estimated *TRA*_C_, *TRA*_N_ and *TRA*_P_, using *V*_max_ and the mass of these elements in the leaf litter (Figure S5). The *TRA*_C_ (*y* = 7.82*x* + 78.29, *r* ^2^ = .35, *p* = .044; Figure 4b) and *TRA*_P_ (*y* = 0.41*x* + 0.32, *r* ^2^ = .79, *p* < .001; Figure 4c) were significantly and positively related to leaf litter P content but not to N and C contents. *TRA*_N_ was not related to any elemental content in the leaf litter.

**FIGURE 5:**
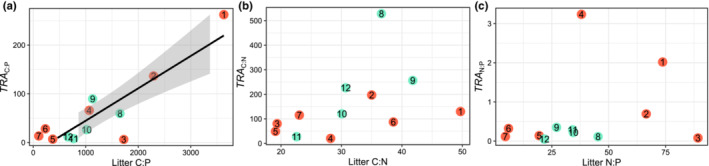
Linear regression between (a) molar C:P ratio of maximal total release amount (*TRA*_*C*:P_) and the C:P ratio of leaf litter biomass, (b) molar C:N ratio of maximal total amount release (*TRA*_*C*:N_) and the C:N ratio of leaf litter biomass and (c) molar N:P ratio of maximal total amount release (*TRA*_*N*:P_) and the N:P ratio of leaf litter biomass. Red circles denote broadleaf leaf litter and aqua blue circles denote coniferous leaf litters. Numbers signify tree species in the order outlined in Table 1 and Figure 1. Significant linear regression and the 95% confidence interval of the linear fitting are presented as solid lines and shaded areas.

Finally, we plotted molar C:P, C:N, and N:P ratios of *TRA* against those ratios of the leaf litter biomasses (Figure 5). The results showed that both the *TRA*_C:P_ and *TRA*_N:P_ were considerably lower than the C:P and N:P ratios of leaf litter, while *TRA*_C:N_ was considerably higher than the C:N ratio of leaf litter. The *TRA*_C:P_ varied considerably (6.4–262.6) among the leaf litter and was higher in Japanese maple and zelkova (1 and 2 in Figure 5a). It was significantly and positively related to the C:P ratio of leaf litter biomass (*y* = 0.065*x* − 15.12, *r* ^2^ = .77, *p* < .001; Figure 5a). The slope of the regression equation indicated that the release efficiency of P was, on average, 15 times higher than that of DOC compared with the elements in leaf litter biomass. Among the leaf litter, *TRA*_C:N_ ranged from 20.43 to 528.31 and was the highest in Japanese hemlock (8 in Figure 5b); *TRA*_N:P_ ranged from 0.05 to 3.24 and was higher in aged oak compared to the other leaf litter species (3 in Figure 5c). However, these ratios were not related to the C:N and N:P ratios in leaf litter biomass, respectively.

## 4 DISCUSSION

When the leaf litter of various tree species, including broadleaf and coniferous species, was soaked in water, we found that P had the highest release efficiency (>50%), followed by C (20%–30%), and N release is the lowest (<10%). This was consistent with the results of previous studies examining the decomposition rate of the leaf litter of various tree species, showing that P was rapidly lost at the early stage of decomposition (McComb et al., 2007; Schreeg et al., 2013). The high release efficiency of P likely stems from the fact that P exists mainly as inorganic orthophosphate or inositol phosphate in plant tissues (Chapin et al., 1990; Yang et al., 2017), which are highly water‐soluble and thus are leached from leaf litter easily.

In this study, we successfully fit the time course changes in *V*_*E*_ to the Michaelis–Menten equation, except for N in some species. However, the estimated *V*_max_ for P exceeded 100% in several leaf litter species including Erman's birch, oak (young), Japanese larch, and hinoki cypress (Table S1). In these species, the P release efficiency from the leaf litter increased almost linearly within our observation period (Figure S3), indicating that 28 days were too short for TDP release to reach the saturation level of release efficiency in these species. Indeed, *k*, the half saturation time, of P seemed to be considerably larger than that of C and N in all species. This trend suggests that the soluble parts of C and N in leaf litter were released more rapidly into the water than that of P, although the fractions of these parts in leaf litter were limited compared to that of P. In the leaf litter, although some of the organic C species, such as sugar, small molecular carbohydrates, and organic acids are water‐soluble, most of the supporting structural carbon, such as lignin and cellulose, are water‐insoluble (Kögel‐Knabner, 2002) and require more time to decompose (Nykvist, 1963). Similarly, N biomass in leaf litter is mainly structural proteins. Although there are highly water‐soluble N such as free amino acids, these are relatively minor in mass (Franklin et al., 2020; Schneider et al., 1998) and mostly recycled during leaf senescence before shedding (Chapin et al., 1990; Nahm et al., 2006). Accordingly, regardless of species, the maximal release efficiency of P was considerably higher than those of C and N in the 28‐day leaching experiment.

Studies based on water immersion of litter and litterbags suggest that the release efficiencies of DOC, TDN, and TDP from deciduous broadleaf leaf litter are higher than those from evergreen coniferous leaf litter (Kiikkilä et al., 2011; Pourhassan et al., 2016; Usman, 2013). Although we found higher DOC and TDN *V*_max_ in broadleaf leaf litter, we did not find a significant difference in the TDP *V*_max_ between the broadleaf and coniferous leaf litter because of the large variations within each of the two taxonomic groups. Interestingly, DOC, TDN, and TDP *V*_max_ (or Max‐*V*) were not positively correlated with the C, N, and P contents, respectively, of the leaf litter (Figure S4a,e,i), suggesting that the soluble fraction of an element does not change proportionally with the elemental content in leaf litter. However, we found that DOC *V*_max_ increased with P content in the leaf litter (Figure 4a). These results are consistent with previous findings that more nutritious leaf litter contains more soluble organic C (Poorter & Bergkotte, 1992). Although the TDP *V*_max_ was not related to the leaf litter P content itself, *TRA*_C_ and *TRA*_P_ were correlated positively with leaf litter P content. These results are consistent with those of previous studies showing that the leaf litter with relatively high P content generally has a high decomposition rate (Osono & Takeda, 2004; Schlesinger & Hasey, 1981). However, neither TDN *V*_max_ nor *TRA*_N_ were associated with the C, N, or P contents in the leaf litter mass and were consistently low relative to those of C and P. As mentioned above, these results may have been affected by the resorption of the water‐soluble N in leaves by trees prior to shedding, regardless of the elemental contents in the leaves.

The C:P and N:P ratios of the maximal total release amounts in leachate, that is *TRA*_C:P_ and *TRA*_N:P_, varied considerably among the leaf litter and were substantially lower than the C:P and N:P ratios of the leaf litter biomass. In contrast, *TRA*_C:N_ was considerably higher than the C:N ratio for the leaf litter biomass. The result implies that the rates and ratios of organic carbon and nutrients released into water cannot be predicted solely through the elemental ratios of leaf litter mass. While *TRA*_C:P_ increased with increasing C:P ratio of leaf litter mass, no such increase was observed for *TRA*_C:N_ and *TRA*_N:P_. Furthermore, as *TRA* is estimated from *V*_max_ and mass contents of leaf litter, and as *TRA*_P_ increased with P content but TDP *V*_max_ was not related to P content, our results indicate that the P release efficiency is relatively less varied among the leaf litter species compared to the C and N release efficiencies. The variations of *TRA*_C:P_ with C:P ratio of leaf litter mass is thus associated with the P content and *TRA*_C_ but not necessarily with *V*_max_ for TDP.

Traditionally, forest leaf litter is viewed as the substrate and nutrient sources for bacterial production in ambient aquatic systems (e.g., Cole et al., 2011; Lennon & Pfaff, 2005; Lindeman, 1942). However, the high P release efficiency supports the idea that leaf litter can also be a supplemental nutrient source for autotrophic organisms, such as algae, whose growth rates are often limited by P supply (Guildford & Hecky, 2000; Sterner & Elser, 2002). Among the leaf litter species, *TRA*_C:P_ was high in Japanese maple and zelkova (Figure 5a). We would infer that high DOC supply from these leaf litter relative to TDP favors bacteria in the competition when bacteria and algae compete for P in aquatic systems (Gurung et al., 1999; Hitchcock et al., 2010; Thingstad et al., 2008). Thus, an increased input of these leaf litter with high *TRA*_C:P_ into ambient aquatic ecosystems may stimulate the heterotrophic microbial production more than the primary production. In contrast, *TRA*_C:P_ of the leaf litter of Siebold's beech, Japanese elm, Japanese red pine, Japanese larch, hinoki cypress, and young leaf litter of oak was considerably lower than 100, although the C:P ratios of these leaf litter mass were higher than 300 in most cases, and, on average, 660 (Figure 5a). Thus, the input of these leaf litter species can stimulate the grazing chains much more than the detrital chains by supporting the primary production in aquatic ecosystems. These possibilities imply that the stoichiometric impact of leaf litter on the food web dynamics in aquatic ecosystems would be better understood if the water‐soluble fractions of elements in the leaf litter species are considered.

Note that in this study, we incubated the leaf litter under a non‐axenic condition, yet we used fresh distilled water to initiate the experiment. Thus, some fraction of the nutrients released from the leaf litter may have been fixed by microbes. However, if these bacteria were suspended in the experimental water, the fractions were included in the measured nutrients (TDN and TDP) in this study. The exception is some C fractions in leaf litter and DOC released may have been respired by microbes. This also implies that the *V*_*E*_ of DOC quantified in this study might be an underestimation. However, assuming this underestimation, it is likely that *V*_*E*_ of DOC would decrease with time. As such a trend was not observed, the effect of microbial respiration on *V*_*E*_ of DOC, if present, would be minimal.

Finally, the stoichiometry of organic and inorganic nutrient release from leaf litter is likely to change depending on both leaf litter species and leaf litter senescence. Observations of oak leaf litter showed that the *V*_max_ of DOC and TDP were lower but that of TDN was higher in the aged leaf litter (Figure 3). This trend may have been caused by C and P mineralization loss and N immobilization through microbial activity on leaf litter during the aging process on the soils (Fellman et al., 2013; Gallardo & Merino, 1992). The immobilized N in aged leaf litter forms N‐rich humus. A fraction of these compounds may have been released later following the immersion into water and decomposition of leaf litter (Gallardo & Merino, 1992). To better understand these processes, it is necessary to examine how the senescence of leaf litter on soil affects the nutrient release efficiencies when it is soaked in water.

## 5 CONCLUSION

In this study, we investigated the release efficiency of DOC, TDN, and TDP of the 12 types of leaf litter from temperate forests. We confirmed that the release efficiency of TDP was the highest during early leaf litter decomposition, followed by that of DOC, and the release efficiency of TDN was the lowest, regardless of the species. More importantly, we found that the maximal release efficiencies and amounts of DOC, TDN, and TDP from leaf litter were highly species‐specific. Furthermore, we found that the DOC:TDP and TDN:TDP ratios of the total maximal release amounts were considerably lower than the C:P and N:P ratios in leaf litter biomass, while the DOC:TDN ratio of total maximal release amounts was higher than the C:N ratio in leaf litter mass. Among the tree species examined, leaf litter of Japanese maple and zelkova released DOC more efficiently relative to TDP, suggesting that these leaf litter species would enhance the bacterial production and detrital chains in ambient freshwater ecosystems. Conversely, leaf litter of Siebold's beech, Japanese elm, Japanese red pine, Japanese larch, and hinoki cypress, and young leaf litter of oak released TDP more efficiently relative to DOC, which would be more beneficial to primary production and grazing chains in ambient freshwater ecosystems. Thus, the present results indicate that changes in the vegetations due to factors such as plantations and climate change can alter the relative importance of aquatic detrital and grazing food chains (Hirama et al., 2022; Prentice et al., 1991).

### 5.1 Significance statement

DOC and TDN and TDP released from forest leaf litter are important energy and nutrient sources that support the production of aquatic food webs. However, the variation of the release efficiencies of dissolved organic matter and inorganic nutrients from leaf litter of different tree species with different stoichiometric characteristics is not well understood. Therefore, we examined the release efficiencies and ratios of DOC, TDN, and TDP of 12 types of leaf litter from 11 tree species. The results showed that the release efficiencies of DOC, TDN, and TDP varied among the leaf litters and that the release efficiency of DOC was influenced by the P content in the leaf mass. Throughout the leaf litter examined, the released DOC:TDP and TDN:TDP ratios were significantly lower than the C:P and N:P ratios of the leaf litter mass, and the released DOC:TDN ratio was higher than the C:N ratio of the leaf litter mass. However, these ratios were highly species‐specific. The results indicate that changes in vegetation with different leaf litter stoichiometry in catchments can alter the structure of detrital and grazing chains in the surrounding aquatic ecosystems.

## AUTHOR CONTRIBUTIONS

**Pei‐Chi Ho:** Data curation (lead); investigation (equal); methodology (equal); writing – original draft (lead); writing – review and editing (equal). **Suzuna Nakajima:** Data curation (supporting); investigation (equal). **Jotaro Urabe:** Conceptualization (lead); funding acquisition (lead); methodology (equal); supervision (lead); writing – review and editing (lead).

## FUNDING INFORMATION

We received the financial support from the Japan Society for the Promotion of Science (JSPS), Grants‐in‐Aid for Scientific Research (KAKENHI; 20H03315 and 23H02548).

## CONFLICT OF INTEREST STATEMENT

The authors declare no conflict of interests.

## Supporting information


Appendix S1

## Data Availability

The data and R codes for analysis are available on Dryad (DOI: https://doi.org/10.5061/dryad.ksn02v79k).
