# Supplementary material for: Stoichiometry of carbon, nitrogen, and phosphorus released from the leaf litter of various temperate tree species
Source: Ecol Evol. 2023 Jul 25;13(7):e10372. doi: 10.1002/ece3.10372 (PMC10368945; doi:10.1002/ece3.10372)
Supplement: Supplementary file 1 — Appendix S1 [file ECE3-13-e10372-s001.docx]

**Supplementary tables and figures**

**Table S1.** Maximal release efficiency (*V*_max_) and half saturation time (*k*) fitted to the Michaelis−Menten equation fitting and observed maximal release efficiency (Max-*V_E_*) during the leaching incubation experiment. Standard deviation of fitted *V*_max_ and *k* and standard deviations of observed maximal releases are presented in parentheses. Parameters with * are statistically significant in non-linear regression (*p* < 0.05).

|  | **Carbon (DOC)** | | | **Nitrogen (TN)** | | | **Phosphorus (TP)** | | |
| --- | --- | --- | --- | --- | --- | --- | --- | --- | --- |
|  | *V*_max_ (%) | *k* (d) | Max-*V_E_* (%) | *V*_max_ (%) | *k* (d) | Max-*V_E_* (%) | *V*_max_ (%) | *k* (d) | Max-*V_E_* (%) |
| Japanese maple | 29.16* (3.91) | 2.12 (1.41) | 27.24  (4.93) | 8.12* (0.75) | 2.31 (1.03) | 7.55  (0.027) | 60.53* (17.11) | 1.89 (2.42) | 57.39  (42.13) |
| Zelkova | 22.36* (3.31) | 1.98 (1.21) | 19.28  (0.27) | 2.87* (0.30) | 1.51 (0.76) | 2.76  (0.31) | 56.26* (14.56) | 4.94 (4.19) | 29.70  (33.69) |
| Erman’s birch | 5.79* (0.61) | 2.67* (1.20) | 6.22  (0.18) | 1.02* (0.22) | 0.36 (0.78) | 1.62  (0.21) | 234.47  (263.52) | 53.46 (83.8) | 78.53  (20.31) |
| Oak (aged) | 16.25* (1.98) | 1.34 (0.89) | 16.81  (0.41) |  |  | 16.38  (14.93) | 39.32*  (5.28) | 2.30 (1.22) | 38.50  (4.68) |
| Oak (young) | 23.78* (2.62) | 0.90 (0.63) | 27.79  (1.31) | 6.78* (2.49) | 9.89 (8.87) | 5.44  (1.35) | 194.12 (170.63) | 41.46 (54.54) | 80.02  (16.01) |
| Siebold’s beech | 20.07* (1.68) | 0.80 (0.46) | 19.66  (1.12) | 6.51* (1.14) | 3.15 (2.21) | 7.34  (0.05) | 24.76*  (1.55) | 0.43 (0.24) | 26.34  (1.43) |
| Japanese elm | 40.40* (4.31) | 0.94 (0.65) | 41.78  (11.84) | 5.92* (0.79) | 1.22 (0.97) | 7.14  (0.23) | 47.96*  (4.48) | 0.34 (0.33) | 55.92  (11.03) |
| Japanese hemlock | 22.52* (3.89) | 5.23 (3.06) | 20.35  (4.76) | 1.14* (0.16) | 0.77 (0.78) | 1.30  (0.016) | 93.44 (44.45) | 20.61 (18.65) | 60.84 |
| Japanese cedar | 17.47* (1.96) | 1.63 (0.93) | 17.06  (2.48) | 2.09* (0.38) | 1.81 (1.53) | 2.35  (0.15) | 33.05*  (7.01) | 2.24 (1.90) | 32.62  (11.05) |
| Japanese red pine | 12.14* (1.66) | 0.93 (0.80) | 14.63  (2.77) | 2.18* (0.65) | 6.43 (5.88) | 2.18  (0.58) | 69.59* (10.70) | 2.22 (1.46) | 71.08  (16.11) |
| Japanese larch | 14.68* (2.28) | 3.06 (2.28) | 14.63  (1.42) |  |  | 8.82  (1.02) | 214.03*  (85.52) | 37.54 (23.13) | 86.12  (3.98) |
| Hinoki cypress | 30.06* (5.59) | 7.26 (3.94) | 23.71  (6.99) | 2.97* (1.06) | 2.95 (4.15) | 3.45  (2.18) | 258.58  (133.78) | 36.39 (28.18) | 99.85  (3.45) |
| Mean | 21.22 | 2.41 | 20.76 | 3.96 | 3.04 | 5.53 | 110.50 | 16.99 | 59.74 |

**Table S2.** Linear regression of maximal release efficiency (*V*_max_) against the C, N, and P contents and molar C:P and N:P ratios of leaf litter mass. Significant regressions are labeled with *.

|  | Intercept | Slope | *R*^2^ | *p* |
| --- | --- | --- | --- | --- |
| Carbon *V*_max_-Litter C | 67.03 | −0.099 | 0.24 | 0.10 |
| Carbon *V*_max_ -Litter N | 31.75 | −0.55 | 0.13 | 0.25 |
| Carbon *V*_max_ -Litter P | 16.75 | 1.91 | 0.35 | 0.044* |
| Nitrogen *V*_max_ -Litter C | 16.27 | −0.023 | 0.059 | 0.45 |
| Nitrogen *V*_max_ -Litter N | 5.47 | −0.0036 | <0.001 | 0.99 |
| Nitrogen *V*_max_ -Litter P | 5.02 | 0.16 | 0.011 | 0.75 |
| Phosphorus *V*_max_ -Litter C | 55.41 | 0.019 | 0.0012 | 0.91 |
| Phosphorus *V*_max_ -Litter N | 33.14 | 1.63 | 0.16 | 0.20 |
| Phosphorus *V*_max_ -Litter P | 71.32 | −3.069 | 0.13 | 0.26 |

**
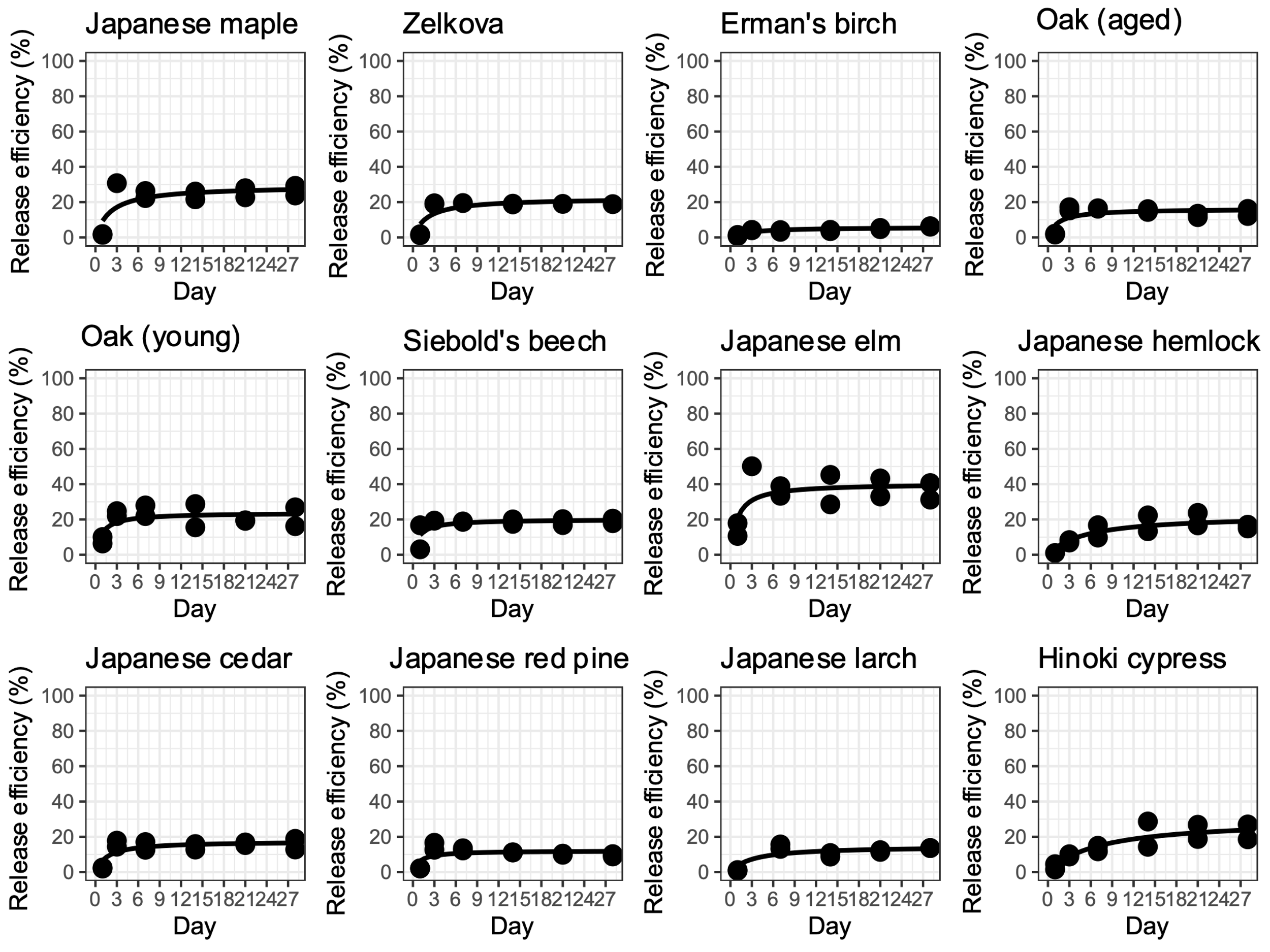
Figure S1.** Michaelis−Menten equation fitting for the release efficiency (%) of dissolved organic carbon (DOC) against leaching time for the leaf litter of 12 types of leaf litter from 11 tree species. Successful non-linear fitting is presented as solid curve.

**
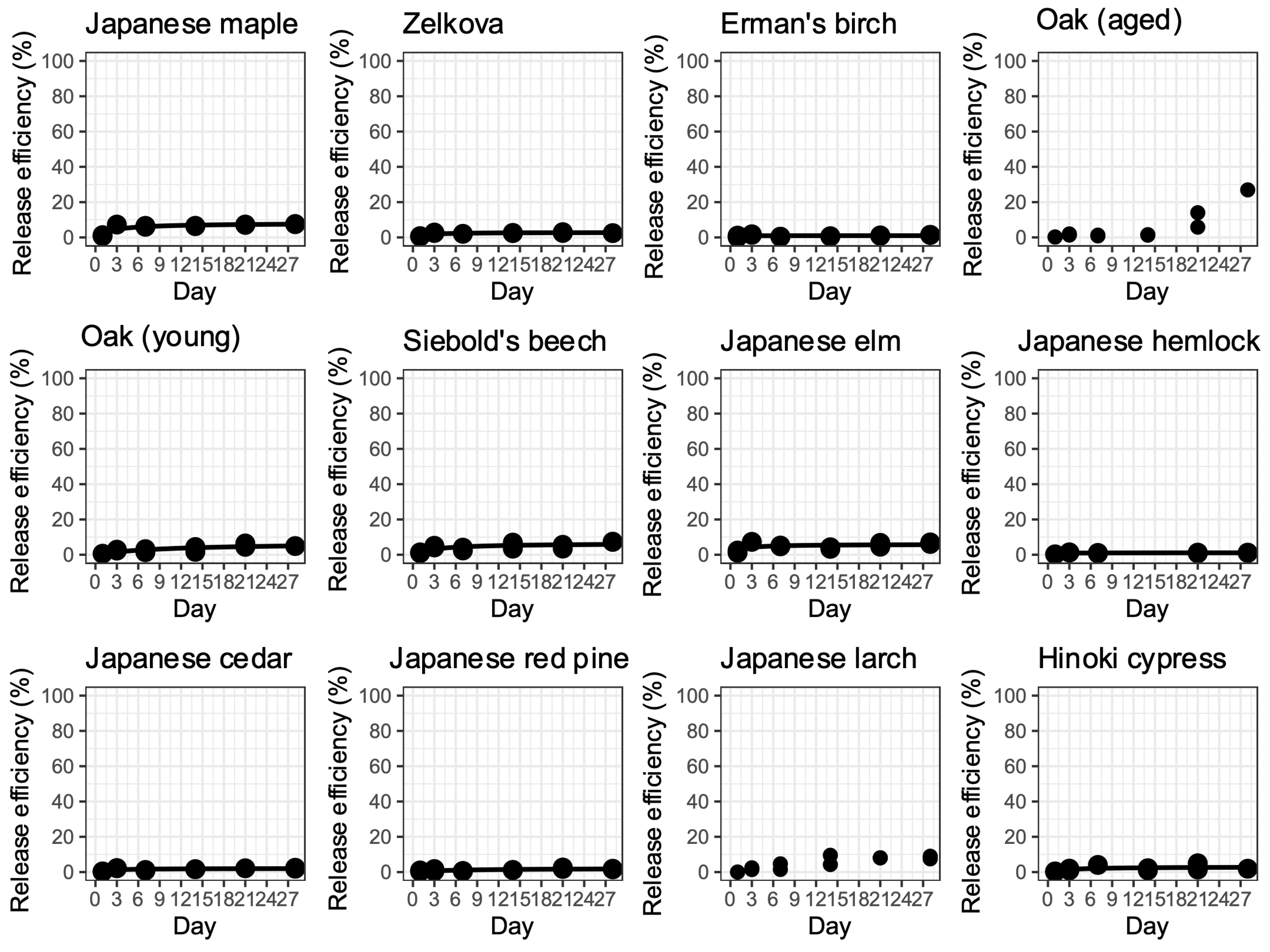
Figure S2.** Michaelis−Menten equation fitting for the release efficiency (%) of total dissolved nitrogen (TDN) against leaching time for the leaf litter of 12 types of leaf litter from 11 tree species. Successful non-linear fitting is presented as solid curve.

**
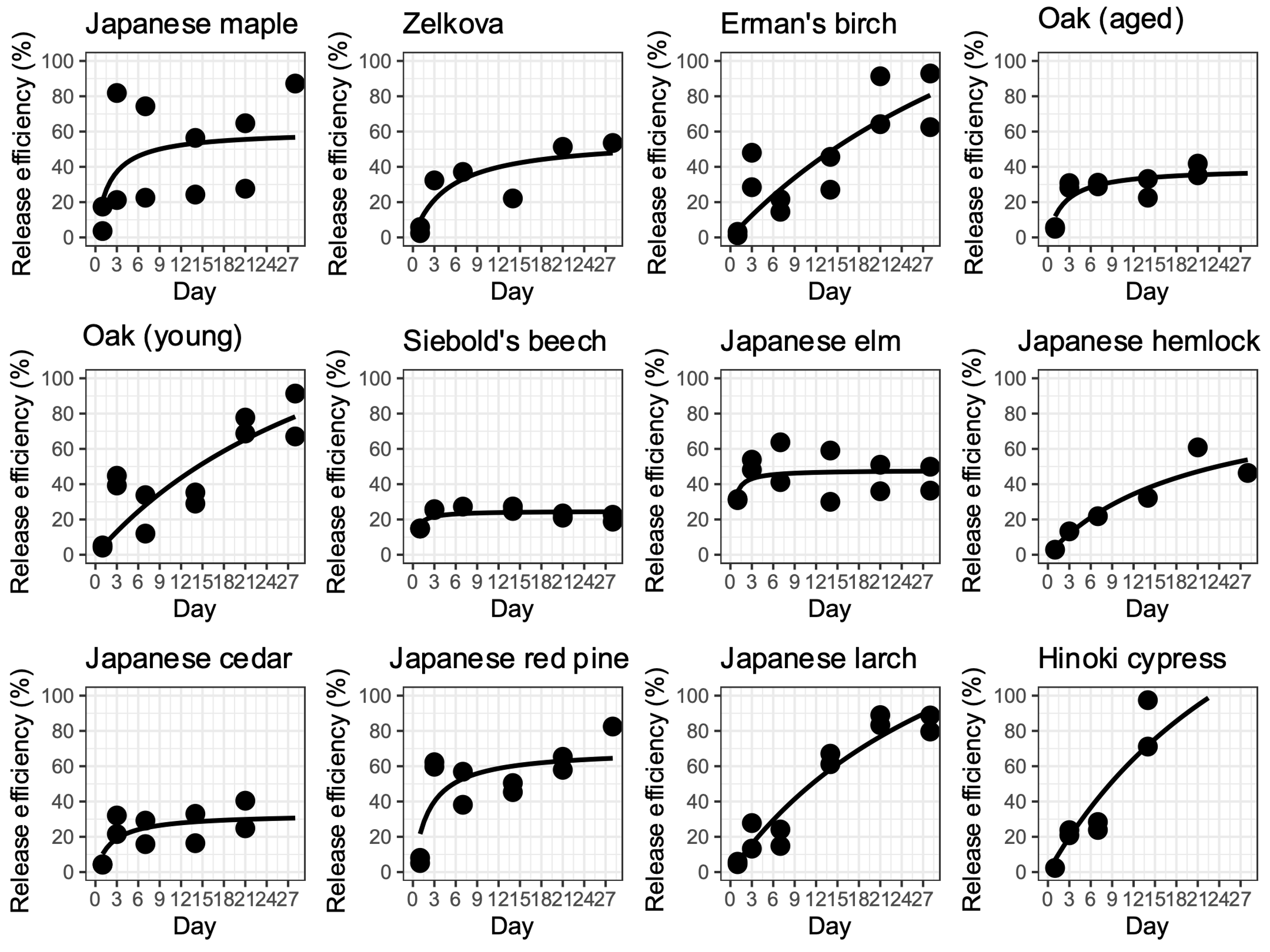
Figure S3.** Michaelis−Menten equation fitting for the release efficiency (%) of total dissolved phosphorus (TDP) against leaching time for the leaf litter of 12 types of leaf litter from 11 tree species. Successful non-linear fitting is presented as solid curve.

**
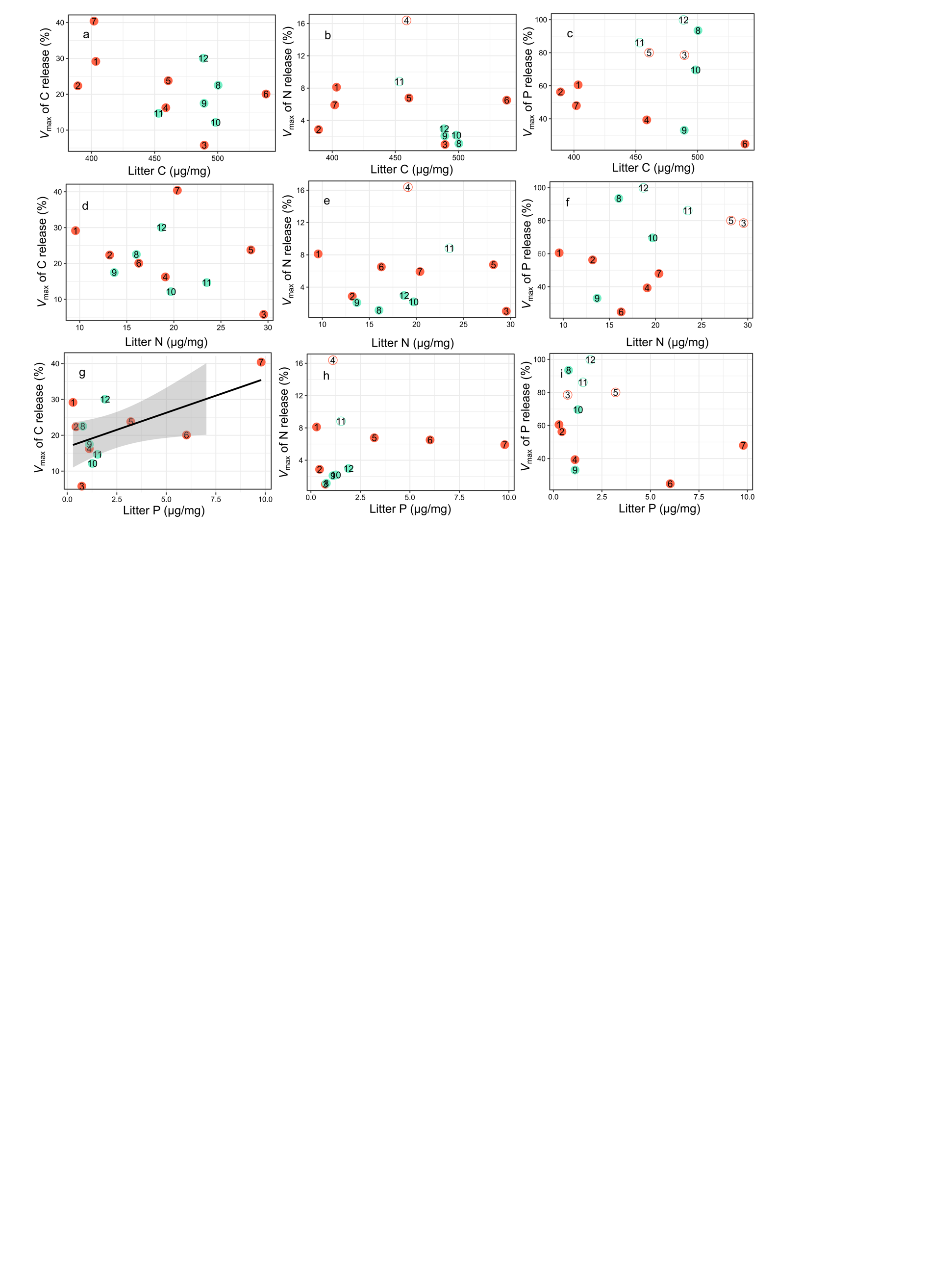
Figure S4.** Linear regression for the maximal release efficiencies (*V*_max_) of dissolved organic carbon (DOC), total dissolved nitrogen (TDN), and total dissolved phosphorus (TDP) against C, N, and P contents in leaf litter. Red circles denote broadleaf and aqua blue circles denote coniferous leaf litters. Open circles in subplots represent observed maximal release efficiency (Max-*V_E_*) from leaf litter leaching experiments that failed to fit the Michaelis−Menten equation (N release from oak (aged) and Japanese larch) or *V*_max_ > 100% (P release from Erman’s birch, oak (young), Japanese larch and hinoki cypress). Numbers refer to the tree species listed in Table 1. Significant linear regression and the estimated confidence interval of the Michaelis−Menten equation fit are shown as a solid line and shaded area.

**
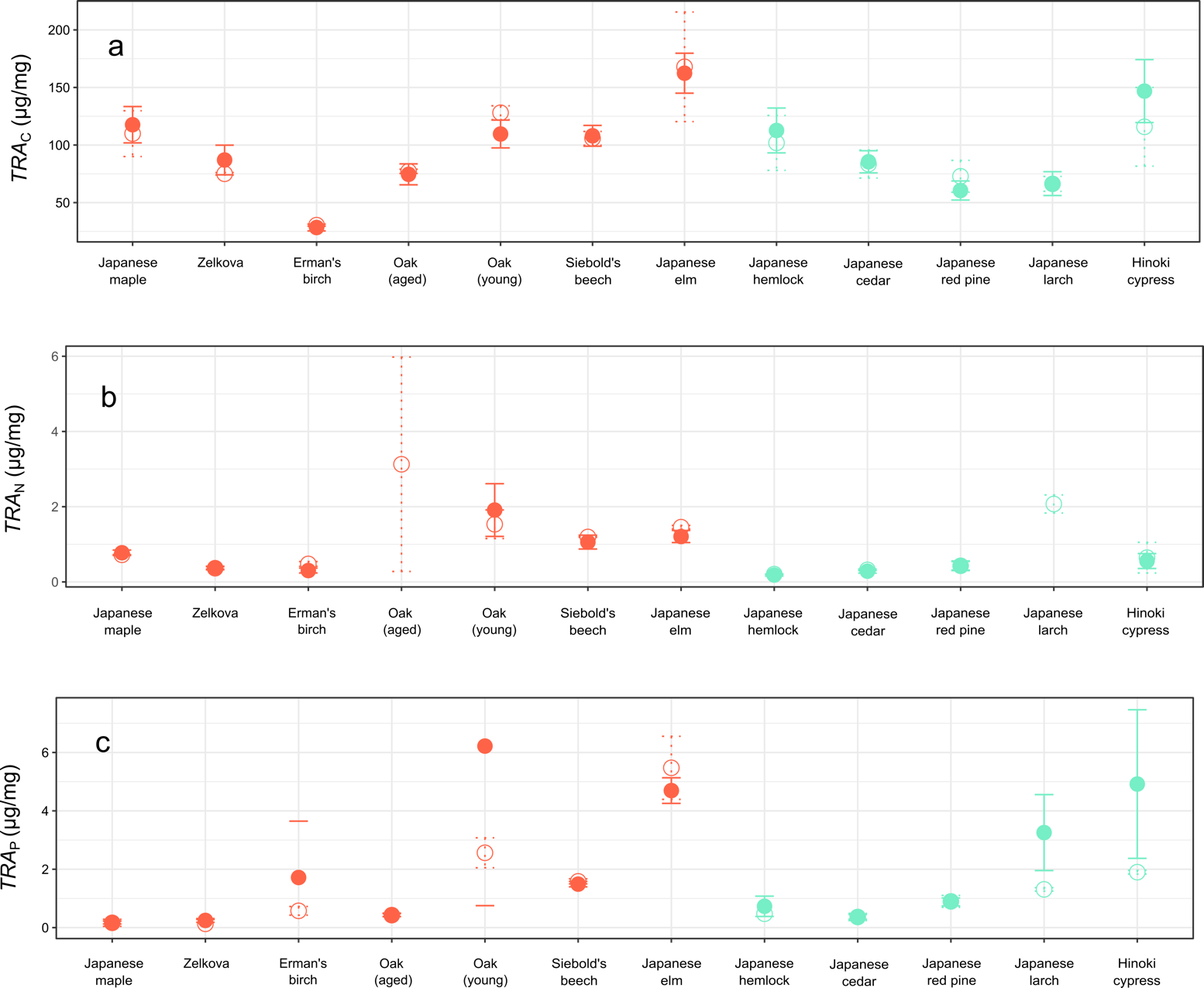
Figure S5**. Maxima total release amount of dissolved organic carbon (DOC) (*TRA*_C_: a), total dissolved nitrogen (TDN) (*TRA*_N_: b) and total dissolved phosphorus (TDP) per mg leaf litter dry mass (*TRA*_P_: c) calculated from leaf litter C, N, and P contents, and *V*_max_ (solid circles and lines) or the observed maximal release Max-*V_E_* (hollow circles and dotted lines) during the 28-day leaching experiment. Red circles denote broadleaf and aqua blue circles denote coniferous tree species.
